# Attentional profiles linked to event segmentation are robust to missing information

**DOI:** 10.1186/s41235-019-0157-4

**Published:** 2019-03-04

**Authors:** Jessica E. Kosie, Dare Baldwin

**Affiliations:** Department of Psychology, University of Oregon, 1227 University of Oregon, Eugene, OR 97403 USA

**Keywords:** Event cognition, Event segmentation, Action processing, Predictability monitoring

## Abstract

Everyday experience consists of rapidly unfolding sensory information that humans redescribe as discrete events. Quick and efficient redescription facilitates remembering, responding to, and learning from the ongoing sensory flux. Segmentation seems key to successful redescription: the extent to which viewers can identify boundaries between event units within continuously unfolding activities predicts both memory and action performance. However, what happens to processing when boundary content is missing? Events occurring in naturalistic situations seldom receive continuous undivided attention. As a consequence, information, including boundary content, is likely sometimes missed. In this research, we systematically explored the influence of missing information by asking participants to advance at their own pace through a series of slideshows. Some slideshows, while otherwise matched in content, contained just half of the slides present in other slideshows. Missing content sometimes occurred at boundaries. As it turned out, patterns of attention during slideshow viewing were strikingly similar across matched slideshows despite missing content, even when missing content occurred at boundaries. Moreover, to the extent that viewers compensated with increased attention, missing content did not significantly undercut event recall. These findings seem to further confirm an *information optimization* account of event processing: event boundaries receive heightened attention because they forecast unpredictability and thus, optimize the uptake of new information. Missing boundary content sparks little change in patterns of attentional modulation, presumably because the underlying predictability parameters of the unfolding activity itself are unchanged by missing content. Optimizing information, thus, enables event processing and recall to be impressively resilient to missing content.

## Significance

In everyday circumstances, people often are unable to give events their undivided attention, due to basic inattentiveness, distractions, or complicated circumstances that obscure access to event information. What happens to one’s processing of events in the face of the resulting missed information? This question seems especially urgent for occupations such as air traffic controllers that hinge upon vigilance to unfolding events. In this research, we examined the impact of missing event content on viewers’ unfolding processing and on their recall of event content. Viewers advanced through slideshows of activity sequences at their own pace, and we measured the time they spent dwelling on slides as an index of their attention. We examined the impact of missing content on event processing by creating a second set of slideshows in which every other slide was deleted. Half of the total content was, thus, deleted in the low-resolution slideshows. Although viewers spent longer on average dwelling on each slide in the context of reduced resolution, their dwell-time patterns were scarcely affected, indicating that event processing is strikingly resilient to missing information. To the extent that viewers compensated for reduced resolution with increased attention to slides, their recall of event content was also relatively unaffected. These findings advance our understanding of the mechanisms underlying event processing, and may help in real-world situations where event-processing integrity is essential. For example, algorithms could be designed that detect when viewers’ inattentiveness is acute enough that their apprehension of event content is seriously compromised.

## Event segmentation is robust to missing information

Events that form the basis of ongoing experience are rapid, continuous, and dynamic. To understand what occurs, predict what might happen next, respond in appropriate ways, and learn from new occurrences, the events require swift and efficient processing in real time. Though events unfold continuously, human experiencers of those events cannot, and do not, give their undivided attention to the streaming activity. Gaps in attentiveness occur for a variety of reasons: objects and actors frequently occlude observers’ access to event information, distance or poor lighting conditions degrade observers’ access, and observers’ attention is inherently mercurial, both because they are frequently distracted or are trying to process multiple events in parallel. However, intuitively, it seems that undivided attentiveness to activity streams is often not necessary for observers to derive the meaning and significance of events. However, precisely how do gaps in access to unfolding information impact event processing?

In recent years, considerable research has focused on one particular component of event processing: segmentation—the processes by which dynamically streaming sensory information is redescribed in terms of discrete event units bookended by event boundaries. Detecting segmental structure within activity streams appears to be a key aspect of fluent event processing that occurs automatically and predicts recall of events (e.g., Baldwin & Baird, 2001; Kurby & Zacks, 2008). In the present research, we specifically investigated how gaps in viewers’ access to event content impacts their sensitivity to the segmental structure of events. To set the stage for this research, we briefly review existing work on event segmentation.

Corporeal existence involves being forever immersed in a continuous sensory flux, but humans somehow derive discrete event experiences from this dynamic sensory flow. Newtson and colleagues (e.g., Newtson, 1973; Newtson & Engquist, 1976) discovered some years ago that human viewers show considerable agreement about the event units they discern within continuously unfolding activity streams, and even largely agree on breakpoints within the temporal flow at which event units begin and end. To offer an everyday example, a typical coffee-making activity stream would be segmented as opening the coffee maker, inserting a filter, adding coffee grounds, pouring in water, and turning the machine on, with event boundaries identified at the initiation and completion of each of these sub-activities. Observers also readily scale their segmentation up or down in terms of the level at which they are asked to identify boundaries. At a coarse level, for example, viewers would likely identify a boundary at the beginning and end of the entire *making a pot of coffee* sequence. At a fine level, an observer could break down *adding coffee grounds* into opening a bag of coffee, picking up a scoop, inserting the scoop into the bag, and so on. Thus, the *making a pot of coffee* sequence can be segmented at various levels of structure. Viewers’ judgments at these different levels of generality are aligned, such that coarse boundaries tend to align with boundaries at the fine level, suggesting that viewers organize event segments hierarchically in their processing (Zacks & Swallow, 2007). Importantly, the extent to which viewers’ judgments of event boundary locations correlate with group agreement is positively associated with event memory (Kurby & Zacks, 2011; Sargent et al., 2013). Individuals who cannot efficiently segment events into discrete units are at risk of having deficits in both memory and the ability to perform everyday activities (e.g., Bailey, Kurby, Giovannetti, & Zacks, 2013; Zacks, Kurby, Landazabal, Krueger, & Grafman, 2016; Zacks, Speer, Vettel, & Jacoby, 2006). Relatedly, explicitly instructing individuals to engage in segmentation enhances their memory of events (Flores, Bailey, Eisenberg, & Zacks, 2017), further underscoring the cognitive importance of the skill at identifying segmental structure within dynamic activity.

One might question whether viewers’ explicit judgments about event boundaries—of the kind just described—are informative about the actual segmentation processes operating as they make sense of events. A variety of tasks providing implicit probes suggest that they are. That is, there is considerable evidence that segmentation processes are indeed operating implicitly when viewers are engaged in observing unfolding activity. While use of the term “implicit” has been debated, we use it here in a non-technical sense to convey that segmentation operates largely outside of conscious intent or awareness. For example, when content is systematically deleted from a film, viewers are faster to detect deletions at event boundaries than deletions occurring within event units (Newtson & Engquist, 1976). During the implicit processing of a film, recall for videos that show only event boundaries is equivalent to that of fully intact videos, while recall for videos that show only within-unit content is significantly worse (Schwan & Garsoffky, 2004). As well, objects that happen to be encountered at event boundaries are better remembered than objects that occur in non-boundary regions, further demonstrating that event boundary content is privileged in memory (Swallow, Zacks, & Abrams, 2009). Neurophysiological activity in specific regions of the posterior cortex and right frontal cortex, recorded via magnetic resonance imaging during passive viewing of events, correlates with participants’ later conscious, explicit segmentation judgments of the same event streams (Zacks et al., 2001). Reaction times also reveal evidence of implicit segmentation. Participants tacitly process an event’s segmental structure as they engage in a change-detection task, suggesting event boundaries intrude on participants’ processing as they carry out unrelated tasks (e.g., Baldwin & Pederson, 2016; Huff, Papenmeier, & Zacks, 2012). Lastly, when viewers advance at their own pace through slideshows depicting unfolding activity streams, they systematically (but unknowingly) dwell longer on slides depicting boundary content relative to slides depicting within-unit content. Moreover, boundary dwelling is extended longer the higher the position a given boundary holds within the event hierarchy (Hard, Recchia, & Tversky, 2011).

Event boundaries clearly are important and influential for overall processing. The precise reason remains a matter of debate. One currently influential account is that event boundaries offer high information value, in the sense that they are points within an unfolding activity at which observers have difficulty predicting what will happen next (Kurby & Zacks, 2008; Ross & Baldwin, 2015; Zacks, Kurby, Eisenberg, & Haroutunian, 2011). As one watches an event unfold, within-segment regions of the action stream are highly predictable. In a *making coffee* event, for example, predictability is high during an actor’s approach toward the kitchen counter; however, once the actor reaches the counter, predictability drops. At this juncture, several possible actions might ensue, even if one is aware that coffee-making is the actor’s goal. Possibilities include opening a cabinet to retrieve a mug, grasping the lid of the coffee can to begin opening it, lifting the top of the coffee pot to insert a filter, or picking up the pot itself to fill it with water. Given the host of possibilities, this is a point within the activity stream where it is important for viewers to pay attention so that they can glean which among these possibilities occurs. Once the next activity is well underway, however, predictability is again high, until the next event boundary occurs. Selectively increasing attention to event boundaries—points within the sensory stream where predictability predictably plummets—enables observers to optimize the efficiency of their event processing. This is important given the rapid pace at which events relentlessly unfold, a processing challenge that Christiansen and Chater (2016) eloquently refer to as the “now-or-never bottleneck.”

Given the evident importance of event boundaries to fluent processing, it seems plausible that missing boundary content would undercut processing. Indeed, there is some existing evidence that supports this hypothesis. For example, Newtson and Engquist (1976) found that viewers had particular difficulty interpreting events when shown a series of images depicting only unfolding within-unit content (i.e., lacking images of boundary content). Additionally, Schwan and Garsoffky (2004) demonstrated that when viewers were shown a video containing only within-unit content, their recall was much worse than their recall for fully intact videos or videos depicting only event boundaries. Do such difficulties occur, at least in part, because missing boundary content undercuts viewers’ ability to segment the activity stream as events unfold?

One of the earlier-described tasks designed as an implicit probe for event segmentation— Hard et al.’s (2011) dwell-time paradigm—seems particularly well suited to answering this question. With this method, viewers use a computer mouse to advance at their own pace through slideshows of images extracted at regular intervals (e.g., one every 500 ms) from videos of ongoing activity. The looking time (aka dwell time) for each slide is derived from the latency between clicks of the mouse. Among other things, dwell-time patterns that emerge with this method appear to be very revealing about observers’ sensitivity to segmental structure as they view activity streams. As described earlier, viewers dwell longer on slides depicting boundary than within-segment content, indicating enhanced attention to event boundaries. As well, dwelling tends to be longer for slides depicting coarse- rather than fine-grained boundary content, reflecting viewers’ sensitivity to hierarchical organization of event segments within the streaming activity. These findings have been replicated across a variety of event types, including full-body activities such as cleaning a room (e.g., Hard et al., 2011), small-scale manual activities such as sleight-of-hand tricks and shoelace tying (Kosie & Baldwin, 2016; Sage & Baldwin, 2014), and even facial displays of emotion (Garrison & Baldwin: Finding emo: Segmenting dynamic emotional displays, under revision). The same systematic dwell-time patterns emerge when preschool-aged children are asked to advance through videos of child-friendly activity sequences such as stacking nesting cups (Kosie & Baldwin, 2017; Meyer, Baldwin, & Sage, 2011; Ross & Baldwin: The role of executive function skills in preschoolers’ event processing and event recall, in preparation ). Dwell-time patterns also predict viewers’ memory for events, such that longer dwelling is positively associated with recall for event content (Hard et al., 2011; Kosie & Baldwin, 2019). This systematic relationship between dwell time and memory provides support for the interpretation that dwell times reflect the amount of attention viewers are devoting to images as they advance through a sequence. Specifically, we suggest that increases in viewers’ dwell times at event boundaries reflect enhanced attention or “increased intensity of information processing” (Hard et al., 2011), which in turn facilitates their memory of the event content.

All in all, the dwell-time paradigm seems to be a valuable tool for implicitly indexing attentional patterns linked with viewers’ event segmentation when they are immersed in making sense of an unfolding activity. Moreover, the dwell-time paradigm seems particularly well suited to investigating the consequences of missing information on participants’ processing of events. For one thing, in previous research the influence of missing event content was assessed only *after* participants had viewed an activity sequence (e.g., Newtson & Engquist, 1976; Schwan & Garsoffky, 2004), whereas the dwell-time paradigm enables an assessment of such impacts as viewers’ processing unfolds across time. In fact, the dwell-time paradigm inherently involves presenting viewers with activity streams that are missing substantial content; that is, only a subset of images from a given activity stream (extracted at regular intervals, such as one every half second) are presented to viewers in the slideshow format. Thus, on the face of it, findings from the dwell-time paradigm would seem to suggest that event segmentation is robust to missing information, even missing boundary content (since extraction at regular intervals randomly deletes some boundary content and some within-unit content). Given that no direct comparison of dwell-time patterns for one-and-the-same event with and without given event content has ever been undertaken, however, it becomes clear with further thought that at present we simply do not know how missing content might affect dwell-time patterns. That is, it is entirely possible that segmentation processes indexed by dwell-time patterns are impacted by the missing content in local, systematic ways. In fact, the dwell-time paradigm provides an ideal way to address directly questions about the consequences of missing information on implicit segmentation. With this paradigm, it is possible to compare the dynamics of viewers’ attention as a given activity unfolds when specific content—whether boundary or within-unit—is present versus absent.

Thus, in the current research, we employed the dwell-time paradigm, inviting viewers to advance at their own pace through a series of slideshows, extracted at 2 frames per second (fps) from digitized videos depicting four different everyday activities (tidying a room, polishing boots, making a cup of coffee, and re-potting a plant). In a second set of nearly identical slideshows, we made one simple change: we systematically removed half of the event content by the simple expedient of deleting every other slide from the 2-fps slideshows to create 1-fps slideshows. Some deleted slides happened to depict boundary content, while others happened to depict within-unit content. This design enabled a number of comparisons of interest.

First, we examined the effect of slideshow resolution on viewers’ dwell times as well as the extent to which typical dwell-time patterns such as a boundary advantage (greater dwelling on slides depicting boundary relative to within-unit content) and a hierarchical advantage (greater dwelling on boundary slides coarse- relative to fine-grained content) were robust to the reduction in slideshow resolution (from 2 fps to 1 fps). We predicted that, overall, dwell times would be longer for 1-fps slideshows but that boundary and hierarchical advantage patterns would be relatively unaffected by the resolution difference. We explain the basis for this prediction in some detail below.

Second, the 1 versus 2 fps design offered a unique opportunity to examine the effects of missing content on the local dynamics of dwell-time patterns. Of central interest was the consequence of missing boundary content. Diverse perspectives seemed plausible regarding the impact of missing content on dwell-time patterns, generating alternative predictions. As described, typically observers display enhanced attention at event boundaries (the so-called boundary advantage) as activity streams past (Hard et al., 2011). One possible account is that this enhancement is a direct response to the event information depicted within the slides themselves. Thus, missing slides (regardless of whether they are boundary or within-unit slides) would have little or no impact on dwell times. In particular, dwelling on slides following missed content would simply reflect viewers’ response to the event information they contain, regardless of whether or not prior event information was available for viewing. As well, for this *simple content-tracking* account, overall, average per-slide dwell times would not be expected to differ for 1-fps relative to 2-fps slideshows. Hard et al. (2011) provided some evidence against this account, in that they found that dwell-time patterns for randomly scrambled slideshows and slideshows viewed backwards do not display a boundary advantage to the same degree as slideshows viewed in chronological order. Based on this evidence, we did not predict this set of outcomes. In any case, however, the present study provided another test of the *simple content-tracking* account within a novel methodological context.

A second possible account is that viewers’ attention responds to physical changes in the motion stream as an activity unfolds, with event boundaries reflecting higher levels of such change than within-unit portions of the activity stream. Newtson, Engquist, and Bois (1977) suggested something like this, proposing that boundaries represent points within a streaming activity where physical change is heightened. In this account, the degree of physical change in the content depicted from one slide to the next would predict the degree of attention devoted to any given slide. To the extent that slide-to-slide physical change is heightened for 1-fps slideshows compared to 2-fps slideshows, the *physical change* account would predict that, overall, average per-slide dwell times should correspondingly be greater for 1-fps than 2-fps slideshows. Likewise, this *physical change* account predicts that the impact of missed content will be directly related to the extent to which physical change is impacted by that missing content. We examined this prediction directly via an objective analysis of pixel changes from each slide to the next in the 1-fps versus 2-fps slideshows. In fact, existing evidence from Hard et al. (2011) militated against the *physical change* account as the sole generator of dwell-time patterns. They found, on the one hand, that physical change was heightened at boundaries, and particularly at coarse boundaries; interestingly, however, boundary and hierarchical advantages in dwell-time patterns persisted even when the physical change was statistically controlled. Thus, the present study provided a valuable opportunity to test the *physical change* account in a novel manner, but we predicted that physical change alone would not account for the pattern of dwell-time findings.

Another contrasting account explains enhanced attention to event boundaries as arising from uniquely informative content right at boundaries. This account seems to be reflected in Hard et al.’s (2011) idea that boundaries may represent conceptual bridges from one event unit to the next. On this *boundaries are conceptually special* account, when a boundary is missing, viewers must infer the important, but lacking, conceptual content, which would seem to be triggered by encountering the next slide following the missed boundary slide. The resulting prediction is that dwell times for within-unit slides following a missed boundary slide (in 1-fps slideshows) should be elevated relative to the identical within-unit slides when the relevant boundary is present (in the corresponding 2-fps slideshows). Put more simply, increased attention that targets boundary slides in the 2-fps slideshows would be transferred to the closest subsequent slides in the 1-fps slideshows.

The final account we considered is the *information-optimization* account we outlined earlier, which presumes that observers increase attention to boundary content to optimize their information uptake just after the boundary, because: (a) predictability seems to plummet directly following event boundaries; hence, (b) information predictably increases at these junctures. In other words, elevated attention at event boundaries in dwell-time slideshows might be due to what boundary slides presage (i.e., a high amount of immediately subsequent information) rather than to what they actually depict. On this *information-optimization* account, missing content would impact dwell times in relation to the degree to which it affects viewers’ ability to identify boundaries within the activity stream. From this perspective, to the extent that boundary identification remains intact despite missing content, the missing content will have little impact on dwell times for slides depicting post-boundary content. Of course, this prediction (no appreciable difference in the boundary-advantage pattern in dwell times for 1-fps vs. 2-fps slideshows) is the same as that made by the first account we considered, the *simple content-tracking* account. Fortunately, however, these two accounts are distinguishable in a different way. As mentioned earlier, the *simple content-tracking* account predicts that, overall, average per-slide dwell times will not differ between 1-fps and 2-fps slideshows. In contrast, the *information-optimization* account would seem to predict longer average per-slide dwelling for low- compared to high-resolution slideshows, given that predictability from each slide to the next would be lower in 1-fps than 2-fps slideshows (and thus, the information value of each slide in a 1-fps slideshow would be heightened).

All in all, a comparison between viewers’ dwelling patterns in relation to slideshows differing in resolution but otherwise matched in content offered a valuable method for distinguishing among distinct accounts of the mechanisms subserving event segmentation. As well, we took the opportunity to examine the extent to which resolution differences impacted participants’ memory of event content. In line with previous research (e.g., Newtson & Engquist, 1976; Schwan & Garsoffky, 2004), we predicted that reducing event content (including event boundaries) would negatively impact recall. Finally, we explored the degree to which dwell-time patterns predicted event memory. In this analysis, we expected to find that participants who displayed longer dwell times at event boundaries would have a better memory of event content (replicating Hard et al., 2011).

## Method

### Participants

The participants were 124 undergraduates (70% female; *M*_age_ = 19 years) from a large university in the northwestern United States. This sample size reflects the amount of data collection that is accomplishable in one term at the University of Oregon. Additionally, in previous research we found that approximately 60 participants are necessary to reveal stable dwell-time patterns. Our sample size is more than twice that number. The ethnic composition of the sample was: 2% American Indian/Alaskan Native, 18% Asian, 3% Native Hawaiian or other Pacific Islander, 2% Black or African American, 80% White, and 4% chose not to respond (participants were allowed to mark more than one category). This ethnic composition was like the general student population at the university. The study’s protocol was approved by the university’s Office of Research Compliance, and all participants received a partial course credit for their participation.

### Stimuli

**Fig. 1:**
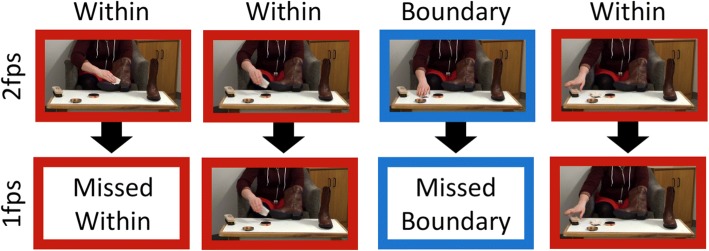
Selected images from the boot polishing slideshow classified as boundary and within-unit slides. This sequence of images illustrates the consequence of removing every other slide from the 2-fps slideshow to create a 1-fps version

The stimuli for the current study consisted of scenarios depicting one of four adult actors engaged in a different everyday activity: polishing a pair of boots, making a cup of coffee, re-potting a plant, and tidying a room. Each of these activities was recorded with a stationary camera at a rate of 30 fps. The total length of the scenarios ranged from 57 s to 112 s. To create slideshows, we extracted still frames from each of the videos at a rate of 2 fps. The resulting slideshows had from 114 to 224 slides. We then created a corresponding set of 1-fps slideshows by systematically deleting all even-numbered (or every other) slides from the 2-fps slideshows.^1^ The length of the resulting 1-fps slideshows ranged from 57 to 112 slides. The resulting set of eight slideshows varied in both the activity they depicted (boot polishing, coffee making, plant potting, and room tidying) and the rate at which frames were extracted (1 fps vs. 2 fps; henceforth resolution; Fig. 1). The natural variation in length across the four activities provided a rough method of controlling for a potential confound between slideshow length and slideshow resolution. The longest 1-fps slideshow contained a similar number of slides to the shortest 2-fps slideshow (Table 1).

**Table 1:** Number of coarse and fine boundaries and within-unit slides for 1-fps and 2-fps versions of each slideshow

Slideshow	Resolution	Number of slides identified as coarse boundaries	Number of slides identified as fine boundaries	Number of slides identified as within-unit	Total number of slides in slideshow
Boot polishing	1 fps	4	21	87	112
	2 fps	5	35	184	224
Coffee making	1 fps	1	14	52	67
	2 fps	9	27	98	134
Plant potting	1 fps	4	13	40	57
	2 fps	5	33	76	114
Room tidying	1 fps	6	16	64	86
	2 fps	11	30	131	172
Total		45	189	732	966

*fps* frames per second

For each of the 1-fps and 2-fps activity sequences, we also created an inverted version of each slideshow by simply flipping images across the horizontal axis. With these inverted slideshows, we hoped to explore the effects of a different viewing format while otherwise equating for slide content, including physical change. Unfortunately, we belatedly discovered that, for non-obvious technical reasons, the inversion process altered the pixelation properties of the inverted slideshows. Upon recognizing this confound, we discarded all data from the inverted slideshows from our analyses.

All participants viewed all four activities with the resolution (1 fps vs. 2 fps) and format (upright vs. inverted) of each slideshow counter-balanced between subjects. Unfortunately, due to the earlier described issue with the inversion process, the data for inverted slideshows were removed from all analyses. Therefore, the data for each participant consisted of dwell times for two slideshows viewed in upright format, one at 1 fps and one at 2 fps.

### Slide classification

We first classified slides from the 2-fps resolution slideshows, as these more densely sampled slideshows contained all possible slides that participants could view. Two expert adult coders first classified slides as occurring at event boundaries or at within-unit junctures of the activity sequences. These coders further defined boundary slides as being coarse- or fine-grained event boundaries. We chose to rely on expert judgments for several reasons. Although prior research documents that participants can reliably identify the approximate location of event boundaries (e.g., Hard et al., 2011; Zacks, Tversky, and Iyer, 2001), variability arises in these judgments for a variety of reasons, including individual differences in understanding the task and in devotion to detail. Expert judgments avoid some of this extraneous variability. Fortunately, participant judgments are typically highly correlated with expert judgments. For example, Kosie and Baldwin (2019) directly compared expert (*N* = 2) and participant (*N* = 22) judgments, finding a point-biserial correlation of .46 between the proportion of participants classifying a given slide as an event boundary and experts’ classification of that same slide (this correlation was .43 when only coarse boundaries were considered and .41 for only fine boundaries). Thus, expert judgments have the benefit of providing boundary judgments that are both representative and precise, which offers a better basis from which to time-lock dwell-time patterns.

Experts defined event boundaries as slides occurring at the earliest moment at which it was obvious a boundary was underway. In the current study, boundaries typically coincided with a change in the actor’s goal state or the objects with which the actor was interacting. For example, the earliest moment at which it became apparent that the actor had finished closing a cabinet in the *coffee making* slideshow was classified as a boundary. Boundaries were classified as coarse if they demarcated large units of structure (e.g., removing coffee grounds from the cabinet and replacing them after they had been added to the coffee maker). Fine boundaries demarcated smaller units of structure that occurred during the activity content between coarse boundaries, such as the moment at which the actor finished twisting the lid on the coffee grounds.

Initial disagreements between expert coders were revised via discussion. Some data suggest that transitions between action units may not be the only cue that signals a boundary to viewers (e.g., 2012; Newtson et al., 1977; Kurby & Zacks, 2012; Zacks, 2004). However, with this in mind, we opt to retain the term “event boundary” in the current study to capture the general phenomenon under investigation. Of the 644 total slides across the four 2-fps slideshows, 155 were classified as event boundaries (30 coarse and 125 fine) and 489 were classified as within-unit slides. As would be expected with varying naturalistic activity sequences, the precise number of event boundaries differed across each of the four slideshows (Table 1).

We next turned to slides in the four slideshows at the 1-fps resolution. Because we were interested in directly comparing responses to slides in the 1-fps slideshows to slides that were present in the 2-fps slideshows, we opted to rely on classifications made at the 2-fps resolution. Therefore, slides classified as boundaries in the 2-fps slideshows that were still present in the 1-fps slideshows were again classified as boundaries (and further classified at coarse and fine levels, corresponding to the identical slide in the 2-fps resolution version). All other slides were classified as within-unit slides. Of the 322 slides across the 1-fps slideshows, 79 slides were classified as event boundaries (15 coarse and 64 fine) and 243 as within-unit slides.

### Pixel change

To assess the physical changes from slide to slide, we used the algorithm described in Loucks and Baldwin (2009).^2^ This algorithm compares the amount of pixel change between any two frames of interest. Here, the frames of interest were individual slides in the slideshow. For the current study, we analyzed the slide-to-slide pixel change separately for each of the four slideshows and across both resolution rates (for a total of eight pixel-change analyses). In these analyses, each individual slide was aligned with the slide immediately following it in the slideshow sequence (e.g., Slide 1 in the boot activity at 1-fps resolution was aligned with Slide 2 in the boot activity at 1-fps resolution, and so on), and the average amount of pixel change across all pixels between these aligned slides was calculated. The resulting value for each slide is a measure of the average amount of pixel change from the immediately preceding slide to the current slide.

### Memory measures

Immediately after viewing each slideshow, participants’ recall memory was assessed using a procedure common in event-processing research (e.g., Hard et al., 2011; Zacks et al., 2006). Participants were asked to list as many actions as they could remember from the slideshow that they had just viewed and to be as accurate as possible. As a guideline, they were informed that they should be able to finish this task in less than 5 minutes and were asked to type their answers. To assess accuracy, we selected a method that has been used to assess recall in previous studies of event processing (e.g., Flores et al., 2017; Sargent et al., 2013). First, we created a list of basic action units, informed by the criteria for A-1 units outlined in Schwartz, Reed, Montgomery, Palmer, and Mayer (1991). Participants’ free-recall responses were scored by a set of three coders, who were instructed to indicate whether participants reported each of the listed actions across the four slideshows. We calculated recall scores by first summing the number of correctly recalled actions for each participant and each slideshow. Next, to account for differences in the number of basic action units across the four slideshows (boot polishing = 50, coffee making = 28, plant potting = 26, and room tidying = 28), we calculated the percentage of correctly reported units by dividing the number of actions correctly recalled by the number of basic action units defined in each slideshow. Approximately 20% of participants’ responses were independently scored by two coders. Coders’ scores were highly correlated, *r*(95) = .98, *p* < .001, 95% CI [.96, .98].

After participants viewed all slideshows and completed the corresponding recall tasks, they completed two other memory tasks. In one task, participants were handed a set of eight still pictures taken from the slideshows they had viewed and were asked to lay out the pictures in the order that they had occurred in the slideshow. In the other task, participants were asked to type out responses to a set of short-answer questions (24 in total, 6 from each slideshow) that pertained to the content of the slideshows they had viewed. Unfortunately, both tasks were subject to ceiling effects; 28–30% of participants in both tasks displayed 100% accuracy on the measures. We, thus, did not explore these memory measures further, though the data are available at the OSF project page associated with this manuscript (https://osf.io/rqmnd/).

### Procedure

After completing the consenting process, participants were informed that they would see action taking place on the computer screen, that the action would be presented in the form of still images, and that they could advance through the images at their own pace by clicking a computer mouse. To familiarize participants with the dwell-time procedure, all participants first advanced at their own pace through a practice slideshow depicting an actor wrapping a present (*N* = 69 slides). After the practice slideshow, participants were informed that they would see more action presented in the form of still images and that they could again advance at their own pace by clicking the mouse. In addition to the instructions given for the practice task, participants were instructed to pay attention to the events, since immediately after the slideshow they would be asked to list as many actions as they could recall and later, they would be asked additional questions about the activities they had viewed. After completing the slideshow and recall tasks, participants completed the order memory task, questions memory tasks, and finally a demographics questionnaire.

### Dwell-time data processing

Participants’ dwell times were recorded using PsychoPy (Peirce, 2007), a user-friendly experimental control system written in Python. Participants’ dwell times indexed the length of time that a given slide was visible on the screen, operationalized as a measure of the latency between mouse clicks. Like most reaction-time data, dwell times were positively skewed and were, thus, first log_10_ transformed to normalize the distribution. Dwell times that were greater than 3 standard deviations (*SD*) above the overall dwell-time log_10_ mean were considered to be outliers, as is typical in analyzing dwell-time data (e.g., Garrison & Baldwin: Finding emo: Segmenting dynamic emotional displays, under revision; Kosie & Baldwin, 2016). In our data, 0.05% of the values were greater than 3*SD* above the mean. These values were then Winsorized (Tukey, 1977), which is the process of replacing outliers with the log_10_ dwell-time value representing 3*SD* above the overall mean. Typically, when more than 10% of an individual’s dwell times were classified as outliers, all data for that participant are removed from the analyses. In the current study, however, no participant met this criterion for exclusion and thus, the dwell-time data for all participants were used in all analyses.

An additional feature of most dwell-time data is the tendency for dwell times to get progressively smaller; that is, observers speed up their dwelling through the slideshow as the activities proceed across time. As a consequence, dwell times for slides at the beginning of slideshows are inflated. To correct for such inflation, which might affect analyses comparing differing slide types (e.g., boundary vs. within; coarse vs. fine vs. within), data were transformed using a residualization procedure. This involved fitting a power curve to participants’ data for each of the four activity slideshows they viewed (significant power curves were observed in 77% of slideshows viewed). The proportion of slideshows fitting a significant power curve did not differ across 1-fps slideshows (73% of slideshows fitting a significant power curve) and 2-fps slideshows (74% of slideshows fitting a significant power curve), *χ*^2^(1, *N* = 248) = 0.02, *p* = .88. We calculated residuals in relation to the power curve for all slideshows across all participants, resulting in dwell-time scores that were sometimes negative. The interpretation remains, however, that longer log_10_ residualized dwell times (henceforth, residualized dwell times) are indicative of longer looking.

## Results

For analyses estimating linear mixed-effects models, we used the lme4 package (Bates, Mächler, Bolker, & Walker, 2015) in R (R Core Team, 2015) with type III sums of squares. Significance for these models was assessed using the lmerTest package (Kuznetsova, Brockhoff, & Christiansen, 2015; Luke, 2017) with Satterthwaite’s approximation for degrees of freedom. To account for variance across subjects and slideshows, all dwell-time analyses included random intercepts for these two variables. Where applicable, linear mixed-effects models included orthogonal contrasts to explore effects of interest.

### Slideshow resolution influences overall dwell time

**Fig. 2:**
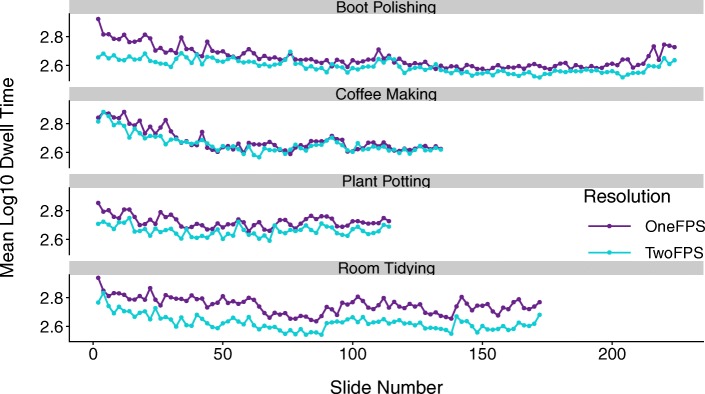
Alignment of dwell-time patterns across 1-fps and 2-fps slideshow versions. Only matched slides, depicting identical content across the two levels of resolution, are included. FPS frames per second

In a first set of analyses, we examined the extent to which slideshow resolution impacted viewers’ per-slide log_10_ dwell times (i.e., prior to residualization but with outliers replaced and dwell times log_10_ transformed to correct for positive skew) as they advanced at their own pace through the slideshows. Average log_10_ per-slide dwell times for 1-fps slideshows (*M* = 2.70, *SD* = 0.24) were significantly higher than those for 2-fps slideshows (*M* = 2.63, *SD* = 0.24), *β* = 0.03, *t*(5.64) = 5.96, *p* = .001. Because the additional content in 2-fps slideshow versions might uniquely influence the observed dwell-time differences related to resolution, we also performed this analysis using only the subset of slides that occurred in *both* the 1-fps and 2-fps slideshows and thus, depicted identical content (henceforth, matched slides; this included all slides in 1-fps slideshows and odd-numbered slides in the 2-fps slideshows). As in our previous analysis, when only matched slides were considered, average log_10_ per-slide dwell times were still longer for slideshows at 1-fps resolution (*M* = 2.70, *SD* = 0.24) relative to slideshows at 2-fps resolution (*M* = 2.63, *SD* = 0.24), *β* = 0.03, *t*(5.51) = 6.02, *p* = .001. In fact, the difference in means for only the matched slides at the 2-fps resolution versus across all slides at 2-fps resolution was extremely small. Despite the overall resolution-related difference in per-slide dwell times, dwell-time patterns were strikingly aligned across 1-fps versus 2-fps versions of a given activity sequence, as can be seen in Fig. 2. As a test of the extent to which dwell-time patterns are aligned across rates of resolution, if we consider only matched slides across the 1-fps and 2-fps slideshows, observers’ log_10_ per-slide dwell times were highly positively correlated, *r*(320) = .72, *p* < .001, 95% CI [.67, .77]. That is, slides that elicited increased dwelling in the 1-fps slideshows were also likely to elicit increased dwelling in the 2-fps slideshows.

These results counter the simple *content-tracking* account: if attention to a given slide simply reflected that slide’s content, we would not expect average per-slide dwell times to differ across 1-fps and 2-fps resolutions, yet they did. These findings also seem inconsistent with the *boundaries are conceptually special* account, which predicts that missing boundaries in the 1-fps slideshows should elicit higher dwelling to subsequent within-unit slides. In particular, *boundaries are conceptually special* predicts that dwell times to a subset of within-unit slides in a given 1-fps slideshow would be high while dwell times to the corresponding within-unit slides in the relevant 2-fps slideshow (e.g., when the boundary *was* present) would be low. Because of this discrepancy, dwell times across the two slideshows would not be expected to correlate strongly, yet they did. In contrast, the strong positive correlation in dwell times across 1-fps and 2-fps versions is consistent with both the *physical change* and *information-optimization* accounts. It is likely that slide-to-slide pixel change (i.e., physical change) is greater in the 1-fps over the 2-fps slideshows, thus predicting longer dwell times. Under the *information-optimization* account, predictability from one slide to the next should be lower for 1-fps versus 2-fps slideshows, with the consequent prediction of longer average per-slide dwell times.

### Dwell-time patterns replicated

In our next set of analyses, we asked whether our results replicated prior research using the dwell-time paradigm. Specifically, we tested for (1) a boundary advantage (longer dwelling on a boundary relative to within-unit slides) and (2) a hierarchical advantage (longest dwell times for coarse boundaries, shorter for fine boundaries, and shortest for within-unit slides). We also explored the extent to which these effects differed across slideshow resolution. This linear mixed-effects model thus included slide type (coarse, fine, and within) and resolution as fixed effects and random intercepts for subjects and slideshows. Additionally, it is relevant to note that in these analyses we considered all slides that were present in 1-fps and 2-fps slideshow versions. That is, slideshows filmed at 2 fps had additional content (including boundaries at both levels of structure as well as within-unit content) relative to 1-fps slideshows. Therefore, in the tests of boundary and hierarchical advantage, we asked about the extent to which such effects were robust to slideshow resolution given all slides that were present in the slideshow (not just matched slides).

This analysis of residualized dwell times yielded a significant boundary advantage (*M* = 0.009, *SD* = 0.12) relative to within-unit content (*M* = 0.001, *SD* = 0.12), *β* = 0.008, *t*(29865) = 6.16, *p* < .001, replicating prior research. Also replicating prior research, average residualized dwell times were greater for: (1) coarse (*M* = 0.017, *SD* = 0.13) over fine-level slides (*M* = 0.007, *SD* = 0.12), *β* = −0.010, *t*(29865) = −3.18, *p* = .001; (2) coarse over within-unit slides (*M* = 0.001, *SD* = 0.12), *β* = −0.018, *t*(29865) = −5.36, *p* < .001; and (3) fine over within-unit slides, *β* = −0.006, *t*(29865) = −3.64, *p* < .001. No significant main effect of resolution emerged in the analysis, *β* = 0.002, *t*(29865) = 1.75, *p* = .08. However, because residualization involves fitting a power curve and calculating deviations from that curve for each participant individually, the process tends to remove differences between individuals, and hence, also group-level differences (which is where resolution differences would occur). Therefore, this discrepancy from the initial set of analyses reported earlier (examining raw dwell times) is not unexpected. As well, no significant interaction emerged in the analysis, *p* = .16, suggesting the boundary advantage persisted across both 1-fps and 2-fps resolutions. All in all, boundary and hierarchical advantage patterns were replicated in these activity sequences, and they were robust to slideshow resolution differences. Regardless of the presence or absence of information (i.e., 1-fps vs. 2-fps resolution), boundaries that remained in the slideshow garnered enhanced attention and did so to a higher degree the greater the granularity of the boundary content.

### Effect of missing boundary content

Thus far, the reported findings appear to rule out the *simple content-tracking* account. Examining the effect of missing boundary content was particularly useful for testing the predictions of the *boundaries are conceptually special* account, as described earlier. Of particular interest was the extent to which missed boundary content might spark enhanced dwelling on the slide depicting content immediately subsequent to the missed boundary. Recall that under the *boundaries are conceptually special* account, when a boundary is missed (as occurs in the 1-fps resolution slideshows), viewers would need to infer the missed content, leading to increased attention to the slide immediately following the missed boundary. Further, we would expect that when that same boundary is present (i.e., in the 2-fps slideshows), dwell times for the boundary slide would be high, but dwell times for the within slide immediately following the (present) boundary would be like other within-unit slides in the event sequence, and hence, relatively low.

Specifically, the *conceptually special* account predicts an interaction between resolution (1 fps vs. 2 fps) and slide type (boundary, within, and within-after-missed-boundary). That is, dwell times should be high for boundaries and low for within-unit slides across both 1-fps and 2-fps resolutions. The locus of the effect, however, should be the difference in dwell times for within-unit slides immediately following boundaries that are missed in the 1-fps slideshows relative to those same within-unit slides following boundaries that are present in 2-fps slideshows. At 1-fps resolution, dwell times on within-unit slides following missed boundaries should be high (as these within slides after missing boundaries are essentially functioning as the opportunity to infer missed boundary content). At 2-fps resolution, on the other hand, the relevant boundary content is present; thus, the very same within slides (that followed missed boundaries in the corresponding 1-fps slideshows) should elicit the reduced dwelling that is characteristic of within-unit content.

**Fig. 3:**
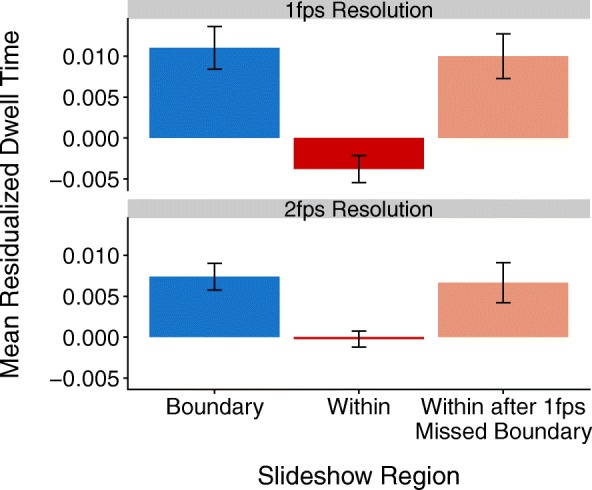
Mean residualized dwell times (± standard error) to boundary slides, within-unit slides, and within-unit slides immediately after a boundary that was missed in the 1-fps slideshows (but present in the 2-fps slideshows). fps frames per second

To explore the influence of missing boundary content, we ran a linear mixed-effects model with fixed effects of slide type (boundary, within, and within after 1-fps missed boundary) and resolution (1 fps vs. 2 fps) and random intercepts for subjects and slideshows. Residualized dwell times on boundaries (*M* = 0.009, *SD* = 0.12) and within-after-missed boundaries (*M* = 0.008, *SD* = 0.12) did not differ, *β* = −0.001, *t*(29859) = −0.38, *p* = .71. However, consistent with the prediction of the *boundaries are conceptually special* account, residualized dwell times for both boundaries and within-after-missed-boundaries were significantly higher than residualized dwell times for within-unit slides (*M* = −0.001, *SD* = 0.12), *β* = 0.011, *t*(29859) = 6.50, *p* < .001 and *β* = 0.010, *t*(29859) = 5.12, *p* < .001, respectively (Fig. 3). The main effect of resolution (1 vs. 2 fps) was not significant, *p* = .49, nor was the interaction between slide type and resolution, *p* = .06.

As an additional test of the *boundaries are conceptually special* account, and despite the non-significant resolution-related effects, we opted also to explore the extent to which residualized dwell times on boundary slides, within-unit slides, and within-after-missed-boundaries differed across the 1-fps and 2-fps resolutions. This analysis tested a key prediction made by the *boundaries are conceptually special* account. Specifically, dwelling on within slides that followed missed boundaries should be higher in 1-fps slideshows (i.e., when the boundary is missed) than on those same slides in 2-fps slideshows (i.e., where that same boundary slide is present), since dwell times for a within slide after a 1-fps missed boundary would be expected to be similar to other within-unit slides in the sequence. In contrast to what the *boundaries are conceptually special* account would predict, there was no significant difference in residualized for within-after-missed-boundaries across 1-fps resolution (*M* = 0.010, *SD* = 0.13) and 2-fps resolution (*M* = 0.007, *SD* = 0.11), *β* = 0.002, *t*(6.43) = 0.61, *p* = .56. Additionally, residualized dwell times for boundary slides did not significantly differ across slideshows viewed at 1-fps resolution (*M* = 0.011, *SD* = 0.13) and 2-fps resolution (*M* = 0.007, *SD* = 0.11), *β* = 0.002, *t*(4.63) = 0.63, *p* = .56, nor did residualized dwell times for within-unit slides (*M*_*1fps*_ = −0.004, *SD*_*1fps*_ = 0.12; *M*_*2fps*_ = −0.0002, *SD*_*2fps*_ = 0.11), *β* = −0.001, *t*(4.39) = −0.99, *p* = .37. This pattern of results held when boundaries were considered separately at the coarse and fine levels of hierarchical structure.

In summary, residualized dwell times to within slides following a missed boundary were elevated relative to other within slides, as the *conceptually special* account predicts. However, a second result clearly militates against the account: when that boundary was actually present (i.e., in the 2-fps slideshows), residualized dwell times for the same within slide were equivalently elevated. This disconfirms the *conceptually special* account’s prediction that dwell times for within slides following missed boundaries will be higher than those for the same slides when the preceding boundary slide is present. The *information-optimization* account, in contrast, is consistent with both these findings, and all findings thus far reported. First, the general difficulty of predicting from one slide to the next would increase as resolution decreases, engendering overall per-slide increases in dwell time for 1-fps compared to 2-fps slideshows. Second, for the *information-optimization* account, boundary slides would be expected to garner increased attention simply because they forecast immediately upcoming low predictability. Given that the predictability structure of the activity depicted in 1-fps and 2-fps slideshows is identical, the account predicts a comparable *pattern* of dwelling on slides across resolution differences (at least, as long as the resolution is high enough that the nature of the activity is discernible). That is, the account predicts—for both 1-fps and 2-fps slideshows—that attention will ramp up in anticipation of boundaries and continue to stay high immediately following boundaries, but will reduce for within-unit slides that occur further away from boundary regions (where predictability correspondingly increases). Our next analyses directly investigated this idea.

### Dwell times reflect boundary regions

In this set of exploratory analyses, we further investigated the *information-optimization* account by examining the time course of residualized dwell times before and after event boundaries. Pre-boundary slides were classified as slides occurring one or two slides before an event boundary, while post-boundary slides were classified as slides occurring one or two slides after the boundary. Thus, the region factor had a total of five levels: (1) two pre-boundary, (2) one pre-boundary, (2) boundary, (4) one post-boundary, and (5) two post-boundary. We explored these region effects separately across the coarse and fine levels of hierarchical structure in a set of two linear mixed-effects models. As in the boundary and hierarchical advantage analyses reported earlier, we included all boundary slides in the slideshows; therefore, slideshows filmed at 2 fps had additional content (including boundaries at both levels of structure as well as within-unit content) relative to 1-fps slideshows.

Overall, we predicted that slides closer to boundaries would elicit increased dwelling while slides further from event boundaries would have reduced dwell times, replicating Hard et al.’s (2011) previous findings that dwell times ramp up as event boundaries approach, and that they decrease thereafter. Under the *information-optimization* account, we might predict that these effects would differ across slide type and rate of resolution. The regions surrounding coarse boundaries are likely less predictable than regions surrounding fine boundaries. Therefore, we might expect that increased dwell times for slideshow regions would persist longer when boundaries fall at the coarse level of hierarchical structure and be more focused at the fine level of structure. Further, we might also predict that it would take longer to resolve unpredictability at the lower 1-fps resolution and therefore, dwell times would remain high for longer after a boundary in these 1-fps slideshow versions. Because of their exploratory nature, the goal of the next set of analyses was simply to characterize the pattern of dwell times before and after event boundaries across 1-fps and 2-fps resolution and coarse- and fine-level slides.

**Fig. 4:**
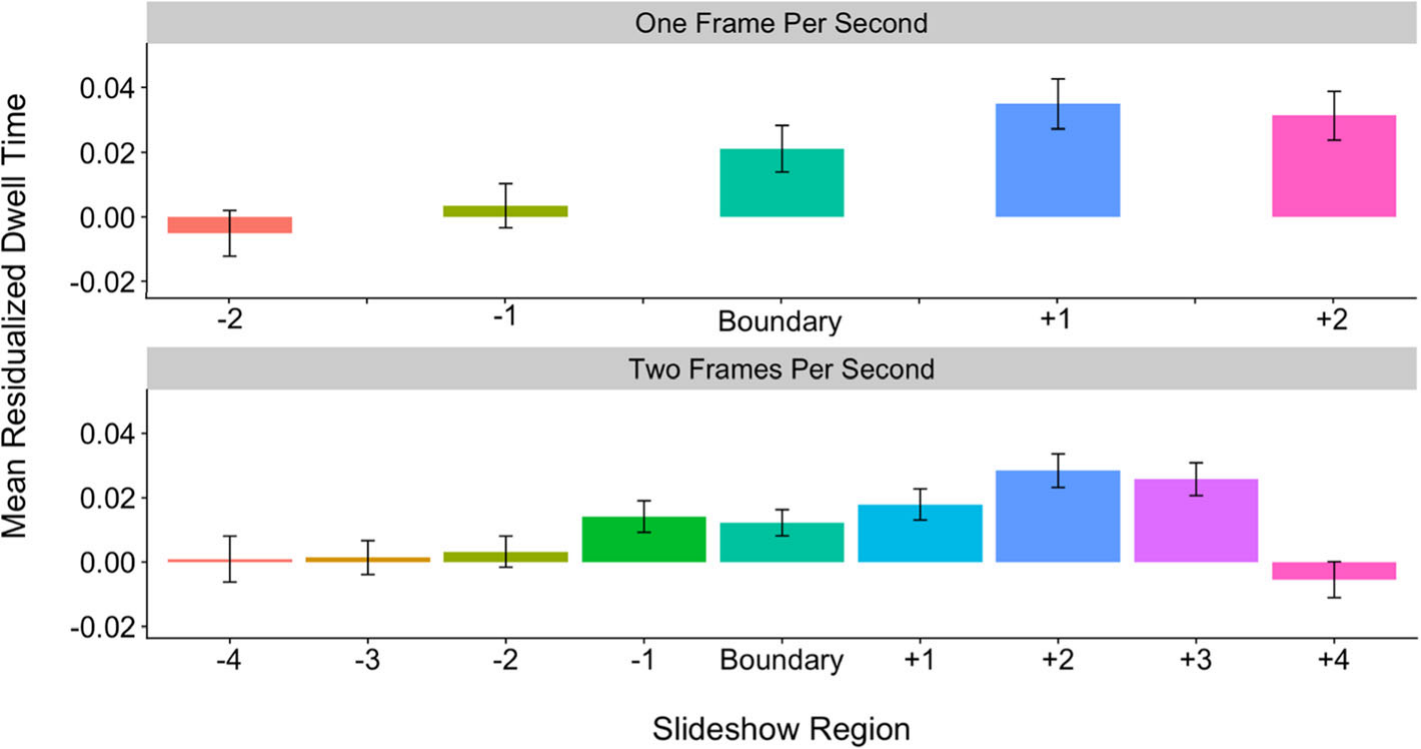
Average residualized dwell times (± standard error) by region, resolution, and slide type for coarse-level boundaries. Slides occurring one and two pre-boundary and one and two post-boundary were included. Bars in the same color represent the same slide content across 1-fps and 2-fps slideshows (i.e., these are the matched slides). fps frames per second

The first linear mixed-effects model included boundary region (two pre-coarse, one pre-coarse, coarse, one post-coarse, and two post-coarse) and resolution (1 fps vs. 2 fps) as fixed effects and subjects and slideshows as random effects (intercepts). For coarse-level event boundaries, the effect of region was best characterized by a linear trend, *β* = 0.02, *t*(5151.11) = 5.37, *p* < .001. While the effect of resolution was not significant, *β* = −0.004, *t*(5.18) = −0.54, *p* = .61, we did find a significant interaction between resolution and the observed linear trend, *β* = 0.008, *t*(5151.10) = 1.92, *p* = .05. Follow-up tests (separate mixed-effects models for 1-fps and 2-fps resolutions) revealed that this interaction was synergistic in nature. While the linear trend was present across both the 1-fps resolution, *β* = 0.03, *t*(1609.47) = 4.18, *p* < .001, and 2-fps resolution, *β* = 0.01, *t*(3425.02) = 3.16, *p* = .002, the effect appeared stronger for slideshows viewed at a rate of 1 fps. As depicted in Fig. 4, for coarse-level boundaries at both 1-fps and 2-fps resolutions, dwell times increased across the two pre-boundary slides, but then remained relatively high across the coarse-level event boundary and the two post-boundary slides.

**Fig. 5:**
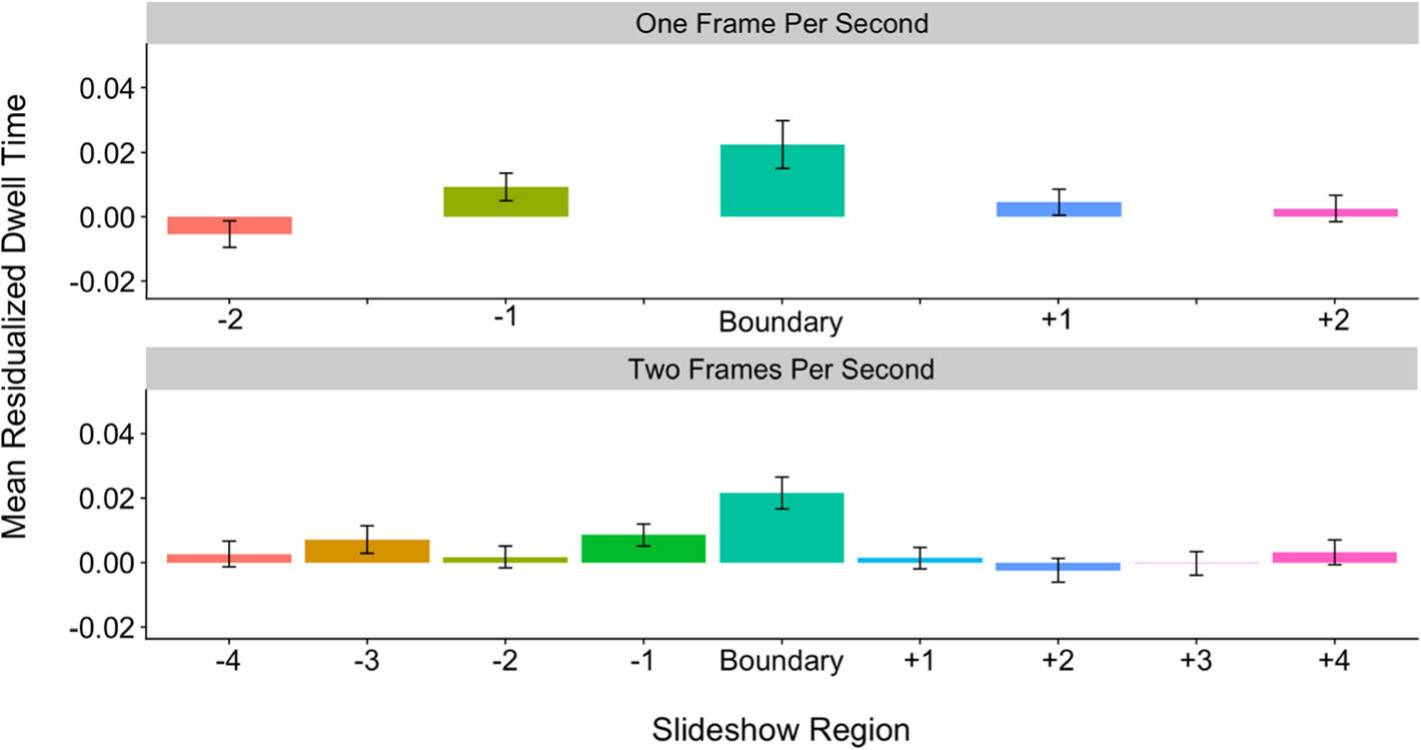
Average residualized dwell times (± standard error) by region, resolution, and slide type for fine-level boundaries. Slides occurring one and two pre-boundary and one and two post-boundary were included. Bars in the same color represent the same slide content across 1-fps and 2 fps-slideshows (i.e., these are the matched slides). fps frames per second

The next linear mixed-effects models focused on fine-level boundaries across slideshows viewed at 1-fps and 2-fps resolutions. We again included fixed effects of boundary region (two pre-fine, one pre-fine, fine, one post-fine, and two post-fine) and resolution and random intercepts for subjects and slideshows. For fine-level event boundaries, the boundary region effect was best characterized by a quadratic trend, *β* = −0.02, *t*(10,249.32) = −5.24, *p* < .001. The effect of resolution was not significant, *β* = 0.001, *t*(5.15) = 0.19, *p* = .86, nor did it interact with boundary region (*p* = .71). As depicted in Fig. 5, dwell times for both 1-fps and 2-fps resolutions increased across the two pre-boundary slides, peaking around the fine-level event boundary, and they began to decline thereafter.

Taken together, these findings largely replicate the results of Hard et al. (2011) and seem to provide further support for the *information-optimization* account of event processing. As predicted under this account, we observed that dwell times ramped up in advance of boundary slides at both coarse- and fine-levels of hierarchical structure. In some cases, especially at the coarse level of structure, dwell times remained high after event boundaries. This, perhaps, reflects the relatively lower predictability junctures represented by coarse-level boundaries. As can be seen in Fig. 4, which includes additional pre- and post-boundary slides, dwell times did begin to decline at about four slides after the coarse-level boundary; perhaps, this is the point at which event sequences become relatively more predictable and thus, requires less attention. For fine-level boundaries, dwell times ramped up before boundaries and began to decline not long after, perhaps because it takes less time to resolve the unpredictability that occurs at fine-level boundaries. Under this account, and as we observed, dwell times should begin declining shortly after the fine-level boundary occurs. While our results thus far generally favor the *information-optimization* account of event processing, we have not yet ruled out the *physical change* account. We next directly tested the extent to which physical change (operationalized here as slide-to-slide pixel change) was related to dwell-time patterns.

### Physical change and dwell-time patterns

Across all slides in 1-fps and 2-fps slideshow versions, we calculated slide-to-slide pixel change using the algorithm outlined in Loucks and Baldwin (2009). Briefly, for each slide, this algorithm compares the RGB values of each pixel to the RGB values of the corresponding pixel in the immediately preceding slide and generates a change value. It was not possible to calculate the pixel change for the first slide in all slideshows (because there was no immediately previous slide); therefore, the first slide was dropped from these analyses.

It seems likely that the slide-to-slide physical change would be heightened for 1-fps over 2-fps slideshows simply due to differences in the rate at which information unfolds across these two levels of resolution. Our first pixel-change analysis directly tested the extent to which physical change differed across 1-fps and 2-fps slideshows. In this analysis, we ran a regression predicting the pixel change from the resolution (1 fps vs. 2 fps). As predicted, the slide-to-slide pixel change for 1-fps slideshows (*M* = 1.11 × 10^7^, *SD* = 4.92 × 10^6^) was significantly larger than for slides in the 2-fps slideshows (*M* = 8.84 × 10^6^, *SD* = 4.14 × 10^6^), *β* = 1.11 × 10^6^, *t*(956) = 7.32, *p* < .001. To ensure that the additional content present in 2-fps slideshow versions was not the sole reason for this difference in pixel change, we next asked whether these results held when we considered only matched slides (i.e., those identical across 1-fps and 2-fps slideshows). We again found that the average slide-to-slide pixel change was larger for 1-fps slideshow versions (*M* = 1.11 × 10^7^, *SD* = 4.92 × 10^6^) than 2-fps versions (*M* = 8.95 × 10^6^, *SD* = 4.25 × 10^6^), *β* = 1.06 × 10^6^, *t*(638) = 5.82, *p* < .001.

Additionally, under the *physical change* account, the degree to which *physical change* is enhanced for a given slide should be directly related to the degree to which *attention* is enhanced for that slide. To explore the relation between physical change and enhanced attention, we ran a regression predicting the mean per-slide residualized dwell time from pixel change. As predicted by the *physical change* account, the pixel change was positively related to the mean per-slide residualized dwell time, *β* = 2.35 × 10^-9^, *t*(956) = 9.83, *p* < .001.

Finally, to explore further the plausibility of a *physical change* account, we tested the extent to which the effects of slideshow resolution and boundary advantage held when controlling for pixel change. We ran another regression analysis predicting the mean residualized dwell time for all matched slides from slideshow resolution, whether the slide was a boundary or within-unit slide, and pixel change. Together, these predictors explained a significant amount of variance (12%) in the residualized dwell time, *R*^*2*^ = .12, *F*(7, 632) = 12.78, *p* < .001. In a model that included pixel change and interactions with resolution, the boundary advantage effect remained significant, *β* = 0.012, *t*(632) = 3.12, *p* = .002, as did the effect of pixel change, *β* = 2.04 × 10^-9^, *t*(632) = 5.44, *p* < .001. The effect of resolution was not significant, *β* = −0.001, *t*(632) = −0.32, *p* = .75. Thus, in contrast to the *physical change* account, the boundary advantage effect accounted for the variance in residualized dwell time above and beyond the effects of resolution and pixel change. However, there was a significant interaction between the boundary advantage effect and pixel change, *β* = −7.7261 × 10^-10^, *t*(632) = −2.06, *p* = .04. Further exploration revealed that pixel change was not strongly related to dwell times for boundary slides in general *r*(154) = .15, *p* = .06, 95% CI [−.01, .30] nor at the coarse, *r*(26) = .19, *p* = .33, 95% CI [−.19, .53], and fine, *r*(126) = .15, *p* = .09, 95% CI [−.02, .32], levels of structure, but it was positively correlated with within-unit slides, *r*(482) = .36, *p* < .001, 95% CI [.28, .44]. None of the additional two- or three-way interactions were significant (*p*’s > .06). Thus, it appears that pixel change does contribute to dwell-time patterns to some extent, but is more predictive of dwell times for within-unit slides. On the one hand, these analyses provide support for the *physical change* account. On the other hand, they yielded doubt that physical change alone provides a complete account of attentional patterns in event processing. All in all, the outcome of the pixel-change analyses generally replicated the findings from Hard et al. (2011) described earlier.

### Overall dwell times predict recall of slideshow content

Immediately after viewing each of the slideshows, participants were given a free recall task: they were asked to list all the actions that they remembered from the slideshows they had just viewed. Because it has previously been demonstrated that removing boundary content negatively impacts event memory (e.g., Newtson & Engquist, 1976; Schwan & Garsoffky, 2004), we first explored the extent to which these results were replicated when activity was viewed via the novel dwell-time procedure. For all participants (*N* = 124), we compared their recall score for the slideshow they viewed at 1-fps resolution to their recall score for the slideshow viewed at 2-fps resolution. Four participants were missing recall data from one slideshow each due to their failure to follow the instructions (these participants reported actions that had occurred in the practice slideshow rather than the coffee (2), boot (1), or tidying (1) slideshow). We ran a linear mixed-effects model including a fixed effect of resolution (1 fps vs. 2 fps) and random intercepts for subjects and slideshows. On average, participants recalled 47% (*SD* = 22%) of the listed actions from each of the slideshows. In contrast to our predictions, however, participants’ recall for items from slideshows they viewed at 2 fps (*M* = 50%, *SD* = 22%) did not significantly differ from slideshows they viewed at 1 fps (*M* = 44%, *SD* = 22%), *β* = −0.03, *t*(5.93) = −0.55, *p* = .61.

Additionally, previous research suggests that observers’ skill in explicit segmentation tasks (e.g., Zacks et al., 2006) and the extent to which they implicitly increase attention to event boundaries (e.g., Hard et al., 2011) positively predicts event memory. In light of such findings, we anticipated that viewers’ log_10_ dwell times, specifically dwell times for event boundaries, would positively predict the number of activities they were able to recall. This prediction was confirmed; viewers’ average log_10_ dwell time for boundary slides in a given slideshow was significantly positively correlated with the number of activities recalled from that slideshow, *r*(242) = .37, *p* < .001, 95% CI [.26, .47]. However, the log_10_ dwell time to within-unit slides was also predictive of the number of actions recalled, *r*(242) = .37, *p* < .001, 95% CI [.26, .48], suggesting that this relation was not unique to boundary slides. As well, when controlling for log_10_ dwell time to within-unit slides, the correlation between boundary slides and number of actions recalled was no longer observed, *r*(242) = .01, *p* = .88. Therefore, this package of findings seems best described as a correlation between average per-slide log_10_ dwell time and recall, *r*(242) = .37, *p* < .001, 95% CI [.26, .48]. Perhaps unsurprisingly, participants who, on average, displayed longer per-slide attention to a given slideshow were able to recall more actions from the activity stream depicted in that slideshow.

Of interest in our final analysis was the degree to which dwell time was predictive of event memory above and beyond the effects of resolution. In this regression analysis, we predicted participants’ memory score for each slideshow that they viewed from their average log_10_ dwell time, the resolution at which they viewed that slideshow, and their interaction. We found that these predictors together explained a significant amount of variance (18%) in recall scores, *R*^*2*^ = .18, *F*(3, 240) = 18.01, *p* < .001. Again, participants’ log_10_ dwell times were predictive of free recall scores, *β* = 0.43, *t*(240) = 6.90, *p* < .001. As in our earlier analysis comparing recall for 1-fps versus 2-fps slideshows, we found that resolution was not a significant predictor of recall scores, *β* = −0.14, *t*(240) = −0.81, *p* = .42. There was also no interaction between log_10_ dwell time and resolution, *β* = 0.03, *t*(240) = 0.54, *p* = .59, suggesting that log_10_ dwell time was a positive predictor of recall across both levels of resolution. In sum, participants’ average per-slide log_10_ dwell time for a given slideshow appears to have been uniquely predictive of the number of actions they recalled and, again, this effect was robust to differences in resolution.

## Discussion

The primary goal of the present research was to examine the impact of missing content on event processing. To do so, we adopted the simple expedient of asking viewers to advance through slideshows depicting continuous everyday intentional activity sequences at their own pace, with some slideshows (1 fps) including only half the slides (every other slide was deleted) of their matched counterpart slideshows (2 fps). Among other things, this design enabled us to examine the efficacy of several different theoretical accounts for why event processing seems to involve viewers selectively targeting event boundaries with enhanced attention.

First, our results replicated key findings reported in other research employing Hard et al.’s (2011) dwell-time paradigm. As viewers advanced through unfolding activity, they displayed systematic attentional enhancement to slides depicting boundary content (the previously observed boundary-advantage pattern), and this was more pronounced the higher the hierarchical level of the boundary content depicted (the previously observed hierarchical-advantage pattern). Additionally, as Hard et al. (2011) also found, viewers’ attention tended to ramp up as boundaries loomed, peaked on (or just after) boundaries, and subsided to low levels two-or-three slides immediately following boundaries. It is perhaps worth noting that these findings point toward the value of thinking in terms of viewers selectively targeting transitional regions between events as uniquely worthy of attention, rather than in terms of paying attention to punctating breakpoints in events, as seems suggested by Newtson and colleagues’ terminology (e.g., Newtson & Engquist, 1976).

Strikingly, the observed patterns in viewers’ attentional profiles were robust to our resolution manipulation. Even though 1-fps slideshows lacked half the actual content of 2-fps slideshows, the boundary and hierarchical advantage patterns were relatively unaffected by the missing content, as was the regional pulsing profile of systematically elevating attention toward the region spanning a slide-or-two prior to boundaries through to two-or-three slides following boundaries. This was true even when it was boundary content that was missing. These findings generally mesh well with research investigating observers’ processing of visual narrative sequences in which certain panels were present versus omitted or blank (e.g., Cohn & Wittenberg, 2015; Magliano, Kopp, Higgs, & Rapp, 2017; Magliano, Larson, Higgs, & Loschky, 2016). However, it was not the case that resolution failed utterly to impact processing at all. In particular, observers tended to display longer average per-slide dwell times when viewing the lower resolution (1 fps) slideshows. In our memory analyses, viewers’ log_10_ dwell times were positively related to recall, above and beyond the effect of resolution. These findings provide further confirmation that dwell-time patterns index attentional modulation.

Together, the findings from this research provide clear evidence that event processing is robust to missing content. As well, the findings provide clarification on the mechanisms likely driving viewers’ systematic modulation of attention as events unfold.

For one thing, that dwell times increased with reduced slideshow resolution ruled out attentional changes occurring as a simple response to the content of individual slides, because for such a *simple content-tracking* account, dwell times for slides that were the same across 1-fps and 2-fps resolutions ought to have been unaffected.

Likewise, the robustness of dwell-time patterns in the face of missing boundary content ruled out a *boundaries are conceptually special* account. That is, this account predicted that when the conceptually special boundary content was lacking, attention to slides temporally subsequent to the missing boundary content should be elevated, but that this should occur only for slideshows viewed at the 1-fps resolution. This did not occur: dwell times for slides occurring immediately after boundaries missing from the 1-fps slideshows were elevated at both the 1-fps and 2-fps resolutions (that is, dwell times immediately following these slides were elevated, regardless of whether the slide was present or absent).

A *physical change* account of attentional modulation fared better, successfully predicting (among other things) a positive correlation between (a) physical change differences across 1-fps and 2-fps slideshows and (b) dwell-time differences across 1-fps and 2-fps slideshows. However, this was a relatively weak relation, suggesting that it was unlikely that physical change alone was responsible for dwell-time patterns. Even more tellingly, as Hard et al. (2011) also found, slide content (e.g., boundary vs. within) significantly predicted dwell times in a regression analysis even when controlling for physical change. Thus, physical change appears to have contributed to viewers’ attentional patterns, but did not by any means fully account for these patterns.

In contrast, an *information-optimization* account appeared generally to accommodate the full pattern of findings that emerged in this research. For this account, the pressure of the “now-or-never bottleneck” (Christiansen & Chater, 2016) incentivizes the compression of complex, continuously unfolding sensory information. Prioritizing attention to target information-rich regions selectively within the sensory flow is one way to achieve such compression. Event boundaries epitomize such highly informative regions, because boundaries forecast unpredictability. As one event or action nears completion, a myriad of possible new events or actions is potentiated. Paying attention during these transitional junctures will, thus, be of high value in terms of information uptake. In contrast, much is predictable during an ongoing event or action and there is little value in attending to these junctures, information-wise. Thus, selectively pulsing attention as event boundaries loom and reducing attention as new events are identified optimizes processing efficiency, potentially leaving more cognitive resources available for other computationally intensive aspects of event processing, such as event categorization, integration and inference, linguistic recoding, memory encoding, planning for future actions, and the like.

A range of findings in the present research coincided with predictions arising from the *information-optimization* account. First, of course, the systematic boundary and hierarchical advantage patterns we observed are to be expected for this account. Second, that viewers tended to dwell longer on average to slides in lower resolution slideshows was consistent with information optimization. In a low-resolution slideshow, content in any given slide tends to be less predictable relative to its predictability in a higher resolution slideshow. Given that the *information-optimization* account predicts greater attention to low predictability information, the account directly predicts longer average dwell times for slides in 1-fps than 2-fps slideshows. Third, information optimization predicts that viewers’ attentional profiles will tend to correspond to the predictability structure of an unfolding activity sequence, with attention (and thus, dwell times) ramping up as boundaries loom and ramping down relatively soon after the next event unit begins to unfold. First observed by Hard et al. (2011), we also observed this characteristic regional pulsing pattern in participants’ dwell-time patterns. As mentioned earlier, this pattern was strikingly robust to missing content in 1-fps relative to 2-fps slideshows, even when boundary content was missing. The *information-optimization* account provides a natural explanation for this resilience: the unfolding activity sequence depicted in 1-fps and 2-fps slideshow counterparts was, of course, identical, and thus the underlying predictability patterns of the two slideshows were identical. Our recall findings made it clear that 1-fps slideshows sampled the unfolding sensory stream frequently enough that viewers could determine what was occurring. In analogy to electrical engineering, a 1-fps extraction rate seems to have met the event-processing equivalent of the Nyquist rate (the minimum rate at which a signal can be sampled without introducing errors) for adequate sampling of a sinusoidal function (e.g., Condon & Ransom, 2016). Thus, the underlying predictability parameters of the activity sequence were in some sense unaffected by the resolution manipulation, and regional pulsing patterns would, therefore, be expected to be likewise relatively unaffected. This then in turn helps to explain why within slides following missed boundary slides received equivalent attention to those same slides when that boundary was present. The sampling rate in 1-fps slideshows was high enough that viewers apparently could anticipate the boundary perfectly well even when it was missing, and thus, dwell times for the relevant within slide were unaffected.

Although findings from the present research both replicated prior findings and provided a host of new information, a number of questions remain for future research. In this initial attempt to uncover the effects of missing content on participants’ online processing, we opted to employ 1-fps versus 2-fps resolutions. Though twice as much information is present in the 2-fps versus 1-fps slideshows, one might still ask whether enough event content is missing in the 1-fps slideshow to find differences in processing compared to events extracted at 2-fps resolution. We believe there are a number of factors, including the type of activity depicted and the actor’s goals for processing, that would influence the answer to this question. Ideally, we would implement a whole continuum of levels of the resolution manipulation, to pinpoint the extraction rate at which event discernibility breaks down and viewers’ attentional profiles presumably lose their robust pattern. Although energy intensive to undertake, this seems a key goal for future research.

In this research we employed four different activity sequences and the findings generalized across them. This is not to say, however, that the same pattern of findings would emerge with a greater diversity of event types. We suspect, in particular, that the processing of activity sequences that proceed at a faster clip, such as those involving delicate manual activity (e.g., knitting or origami construction), might well fail to be resilient to a 1-fps extraction rate and that the 1-fps versus 2-fps manipulation could yield quite different patterns of attention. Conversely, if one’s goal was simply to understand an event (such as cleaning the house) at a very coarse or gist level, an extraction rate even lower than 1 fps would likely enable one to identify that the actor cleaned the bathroom, did the dishes, and then mopped the floor. Also of particular interest is the extent to which the same degree of missing content might affect the processing of novel event sequences despite leaving the interpretability of relatively familiar event sequences unaffected. In other words, higher resolution information may be essential for the interpretability of novel event sequences, but may be much less crucial when the predictability structure of event sequences is already well known.

In this research, missing content was engineered via the simple strategy of deleting every other slide from a 2-fps version of a given activity sequence. Of course, however, in naturalistic event processing, information is missed for a variety of reasons (e.g., random inattention, occlusion of relevant information, poor viewing conditions, and distracting conditions) and likely would not occur on a regular time schedule. Thus, the present findings provide an unknown degree of approximation to the impact of missing content in the wild. This is a very real concern when we consider the possible negative implications of such inattention in scenarios such as air traffic control or war-fighter vigilance. At present, it is not safe to generalize these findings to such scenarios. In addition, our findings appear to suggest that a mild degree of inattention or distraction is unlikely to undercut event processing radically, and they hold promise that one day it may be possible to develop an algorithmic detector for when levels of inattention exceed a criterion of discernibility for the relevant event types at issue.

Although our findings suggest that event segmentation and recall are relatively robust to missing content, especially when heightened attention compensates for low resolution, individuals might well differ in their ability to compensate in this way. Those with executive difficulties (e.g., those with attention deficit hyperactivity disorder or dementia) might be at risk in this regard (e.g., Ross, Child, & Baldwin, 2016; Zacks et al., 2006), rendering their processing considerably more fragile than what we observed here under conditions of missing or degraded event content. This is another interesting avenue for future research.

The *information-optimization* account that gains support from the current research also makes general theoretical contact with other accounts of event and narrative comprehension, such as Gernsbacher’s structure building framework (e.g., Gernsbacher, 1995), although the present research provides unique specifics regarding the role of predictability monitoring in event processing. However, the whole existing body of work on event processing, including the current research, leaves many questions unanswered. One such question is the extent to which event processing should best be characterized as pursuing a segmental analysis of sensory information as it streams past, with information optimization a useful strategy, or whether information optimization is itself the goal of processing, with segmental analysis arising as a useful by-product. Answers to this and many other fundamental questions await further investigation.

## Conclusion

When asked to advance at their own pace through slideshows depicting continuously unfolding activity sequences, viewers displayed a set of systematic attentional patterns indicative of efficient optimization of unfolding information. They selectively attended to information-rich content, and avoided attending to content that was highly predictable. Strikingly, removing half the content from the slideshows had little effect on these attentional patterns. These findings both underscore the resilience of event processing and point to information optimization as the best available account of such resilience.

## Abbreviations

fps: Frames per second
OSF: Open Science Framework
*SD*: Standard deviation

## Footnotes

1 There are a number of possible ways to undertake the full versus reduced-content manipulation. For example, one might choose to create a third set of slideshows at the 1-fps resolution by extracting all odd-numbered slides to control for differences in the effect of resolution that might be explained solely by slide content. We opted not to undertake this comparison because it would require a corresponding increase in sample size. Our inclusion of four different action scenarios should serve to ameliorate concerns that differences in resolution are driven solely by features unique to specific slides.

2 Following Loucks and Baldwin (2009), we used the following algorithm to calculate pixel change:

$$ \sum \limits_{i=1}^h\sum \limits_{j=1}^w\sqrt{{\left({R}_{Cij}-{R}_{Sij}\right)}^2+{\left({G}_{Cij}-{G}_{Sij}\right)}^2+{\left({B}_{Cij}-{B}_{Sij}\right)}^2} $$

In this algorithm, *R*, *G*, and *B* represent the red, green, and blue colors of a pixel, *C* and *S* denote the change frame (current frame) and standard frame (previous frame), *i* and *j* represent the coordinate value of the pixel, and *h* and *w* represent the height and width of the frame in pixels.
